# Improving the Prediction of Persistent High Health Care Utilizers: Retrospective Analysis Using Ensemble Methodology

**DOI:** 10.2196/33212

**Published:** 2022-03-24

**Authors:** Stephanie N Howson, Michael J McShea, Raghav Ramachandran, Howard S Burkom, Hsien-Yen Chang, Jonathan P Weiner, Hadi Kharrazi

**Affiliations:** 1 Applied Physics Laboratory Johns Hopkins University Baltimore, MD United States; 2 Center for Population Health Information Technology Johns Hopkins School of Public Health Baltimore, MD United States

**Keywords:** persistent high utilizers, ensemble methodology, utilization, prediction, machine learning, population health analytics, retrospective, observational

## Abstract

**Background:**

A small proportion of high-need patients persistently use the bulk of health care services and incur disproportionate costs. Population health management (PHM) programs often refer to these patients as persistent high utilizers (PHUs). Accurate PHU prediction enables PHM programs to better align scarce health care resources with high-need PHUs while generally improving outcomes. While prior research in PHU prediction has shown promise, traditional regression methods used in these studies have yielded limited accuracy.

**Objective:**

We are seeking to improve PHU predictions with an ensemble approach in a retrospective observational study design using insurance claim records.

**Methods:**

We defined a PHU as a patient with health care costs in the top 20% of all patients for 4 consecutive 6-month periods. We used 2013 claims data to predict PHU status in next 24 months. Our study population included 165,595 patients in the Johns Hopkins Health Care plan, with 8359 (5.1%) patients identified as PHUs in 2014 and 2015. We assessed the performance of several standalone machine learning methods and then an ensemble approach combining multiple models.

**Results:**

The candidate ensemble with complement naïve Bayes and random forest layers produced increased sensitivity and positive predictive value (PPV; 49.0% and 50.3%, respectively) compared to logistic regression (46.8% and 46.1%, respectively).

**Conclusions:**

Our results suggest that ensemble machine learning can improve prediction of care management needs. Improved PPV implies reduced incorrect referral of low-risk patients. With the improved sensitivity/PPV balance of this approach, resources may be directed more efficiently to patients needing them most.

## Introduction

Population health management (PHM) programs regularly classify patients by estimated risk of high health care utilization such as hospitalization [1]. The classification process enables PHM programs to allocate their limited resources according to the patients’ anticipated needs [1,2]. Higher-risk patient groups, if identified correctly, can receive effective interventions such as care management program enrollment to reduce utilization and improve outcomes [2]. Additionally, when utilization and costs are successfully contained for high-need patients by proactively preventing undesired outcomes, PHM programs can better allocate the remaining resources to improve the outcomes of other patients [3].

The set of high-risk patients frequently changes over time, with most patients being high-risk for a short term [4,5]. However, some high-risk patients use health care resources persistently for an extended period (eg, more than 24 months) [4-6]. These persistent high utilizer (PHU) patients generally constitute a small segment of the overall patient population but use a considerable proportion of resources in long term [4-6]. Despite the variety of approaches taken to characterize PHUs, such as adjusting for type of utilization, total costs, number of chronic conditions, and other factors, predicting who becomes a PHU has remained an analytical challenge [7-11].

Past studies have applied several analytical approaches to identify and predict PHUs in different patient populations. These approaches range from traditional regression methods (eg, logistic regression) [4-8] to complex machine learning techniques (eg, gradient boosting and neural networks) [9-11]. Nonetheless, due to the small number of PHUs in a patient population (often less than 5%), most studies have suffered from either oversensitive models or excessive false predictions of high utilization [3,5]. Thus, the challenge of achieving simultaneously useful levels of sensitivity and positive predictive value (PPV) in PHU prediction models has limited their application in practice [12].

To address the methodological challenges in predicting PHUs, this study tests an ensemble approach to balance the sensitivity and PPV of PHU forecasting at practical levels. The ensemble approach uses a mix of machine learning methodologies to improve both the sensitivity and PPV of PHU predictions at the same time. Using insurance claims data of a large patient population, this study compares the ensemble approach to single models, a baseline model, and a more advanced predictive model.

## Methods

### Overall Aims and Definitions

The overall goal of our study was to assess the value of ensemble methodology for achieving required levels of sensitivity and PPV for PHU prediction. Our analysis aimed to provide a methodology to optimize the tradeoff of highly sensitive and highly specific predictive models of PHUs using an ensemble approach.

We defined a PHU as an individual who remained in the top 20% of highest health care costs for 4 consecutive 6-month periods (ie, total of 24 months after the base period) [4]. Health care costs were defined as the sum of costs covered by the insurer and the patient’s out-of-pocket costs [4].

### Data Source and Preparation

We performed a retrospective analysis of the Johns Hopkins Health Care insurance claims data collected between 2013 and 2015. We applied the Johns Hopkins Adjusted Clinical Groups (ACG) software to the claims data to prepare the data for analysis [13]. We categorized the diagnostic codes into higher-level diagnosis groupings called expanded diagnostic clusters (EDCs), and we grouped medication data into Rx-defined morbidity groups (RxMGs) [4,13]. EDCs and RxMGs have been substantially validated in past studies and are routinely used for risk stratification in practice [4,14].

### Study Population

**Figure 1:**
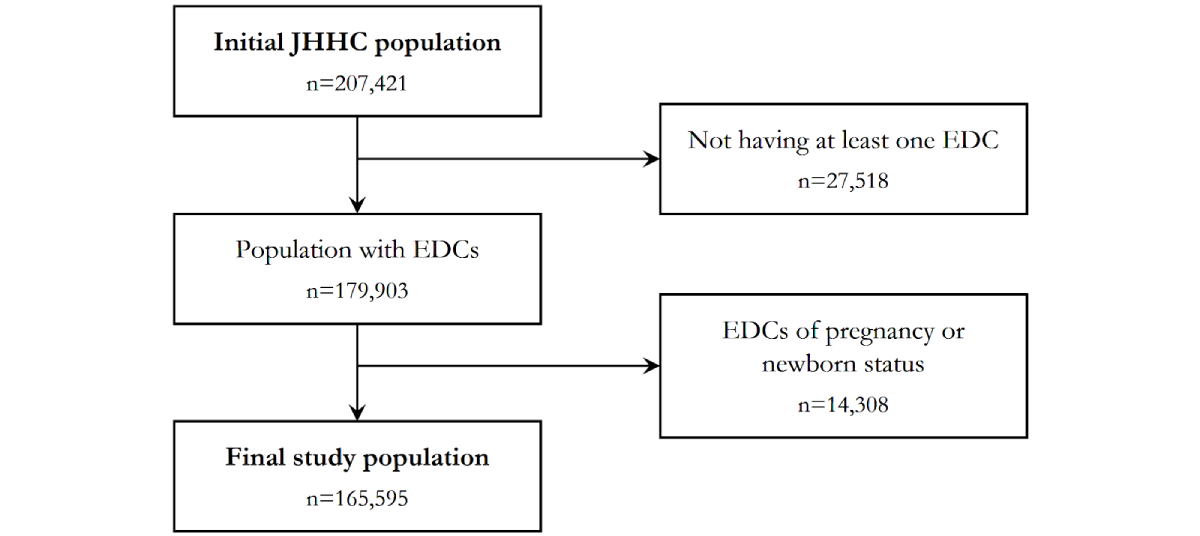
Selection process of the study population. JHHC: Johns Hopkins Health Care; EDC: expanded diagnostic cluster.

Johns Hopkins Health Care claims data included 207,421 patients with at least 1 record in 2013 and at least 2 years of continuous enrollment between 2013 and 2015 (Figure 1). First, 27,518 patients with missing EDC diagnosis codes were excluded, since EDCs were used to predict PHU status within the population. Second, 14,308 patients with EDC codes indicating pregnancy/newborn status were removed, as the anticipated high utilization incurred by these patients are different from PHUs. The final study population included 165,595 patients (Figure 1).

### Predictors and Outcome

Predictors (ie, independent variables) included demographics, EDCs, RxMGs, and other health utilization variables (eg, hospitalization) generated by the ACG system. Many of these predictors, including all EDCs and RxMGs, are categorical variables [13,14].

The outcome of interest, a binary variable, was whether a patient became a PHU after the base year (ie, incurred health care costs in the top 20% of all patients over 4 consecutive 6-month periods).

### Statistical Approach

#### Ensemble Methodology

PHUs constitute a small fraction of the patient population, hence producing a large class imbalance (ie, most patients are non-PHUs). A common issue with single model prediction of highly imbalanced classes is compromising PPV in favor of higher sensitivity. For example, a single predictive model of PHUs may result in many false positives (ie, low PPV) if aiming to capture all PHUs (ie, high sensitivity). However, ensemble models provide a unique opportunity to increase both PPV and sensitivity by combining substantially different predictive models. We hypothesized that an ensemble approach can predict PHUs with both a manageable PPV and an optimal sensitivity compared to basic and advanced single model predictions.

We assessed several machine learning algorithms to predict PHU status among the study population. We also evaluated the performance of the ACG system, a comprehensive regression-based risk stratification tool commonly used in PHM practice [13]. As hypothesized, each of these algorithms yielded average levels of PPV, and we used an ensemble methodology to boost the overall PHU prediction performance.

Ensemble methods take inputs from multiple models and combine the outputs in various ways to strengthen prediction results [15]. In classification problems with imbalanced classes, ensemble methods perform well because multiple models can contribute individual strong features to the overall prediction [16]. Since PHUs make a fraction of the total population, the occurrence of a PHU in the data can be considered an anomaly [4]. Sometimes referred to as anomaly detection, the supervised machine learning problem of classifying PHUs is known as the imbalanced class problem, where the majority class (ie, non-PHUs) is much more prevalent than the minority class (ie, PHUs).

**Figure 2:**
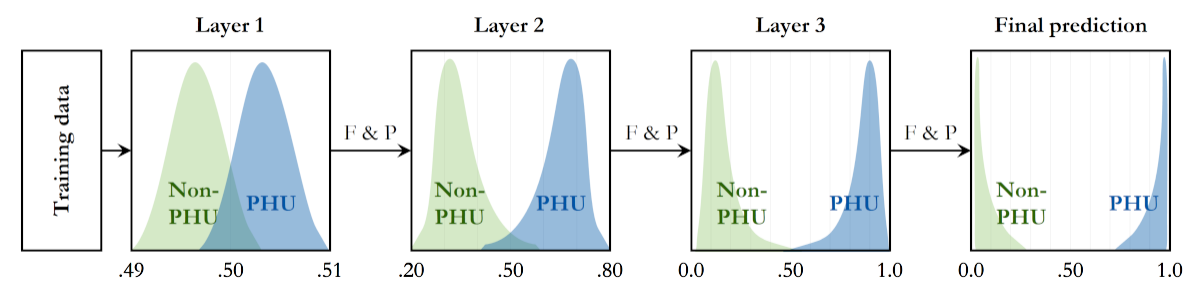
Stacking ensemble architecture. F&P: feature selection and predictions; PHU: persistent high utilizer; non-PHU: nonpersistent high utilizer.

We chose the stacking ensemble model rather than the voting ensemble approach. The stacking ensemble model uses a metaclassifier to aggregate the results, but the voting ensemble model needs user-specified weights to combine the classifiers, hence adding an unpractical step [15]. Thus, for this problem space and our data set, we chose the stacking ensemble. Stacking ensemble methods often use multiple model layers and a final prediction model layer. Each layer makes predictions on the input space given. We also used an additional parameter, feature propagation. This technique allows the passing of both features and predictions through each layer of the ensemble [15]. Figure 2 depicts the overall structure of our ensemble methodology and schematically shows how multiple layers can improve PPV and sensitivity simultaneously (Figure 2).

#### Ensemble Component Model Selection

The models selected as the layers in the ensemble method were chosen using common techniques, namely assessment of common classification algorithms and random search cross-validation for parameter tuning. Typically, machine learning models are assessed for performance and generalizability. Generalizability is difficult to quantify without large unseen data sets available for testing, but a common technique to test for overfitting is k cross-fold validation. This technique tests the machine learning model against many different subsets of data and then calculates an average of all tests. For classifying PHUs, generalizability is fundamentally important because future populations tested through these algorithms will have a large variety of differences, including demographic profiles and medical conditions. Accordingly, we employed several techniques to tune the performance and generalizability of individual models before constructing the layers of the stacking ensemble [15-17].

First, we incorporated an algorithm known as complement naïve Bayes (CNB), which often produces highly sensitive predictions when classes are imbalanced [18]. The CNB model is derived from standard multinomial naïve Bayes [18]. This model has 3 main parameters, alpha, fit prior, and norm [18]. Alpha is a Laplace smoothing parameter that adjusts the shape and fit of the multinomial distribution. This parameter shifts and forms the training distribution to characterize the multidimensional space of the data. Fit prior refines class identification when only a single class is found in the training set, which can easily occur since PHUs occur infrequently in the data set. Fitting the priors of the classifier ensures that the majority class (ie, non-PHUs) still has some probability of not occurring, even though no other class is present in the training data. The norm parameter determines whether the training involves a second normalization of weights, an additional measure to bolster the performance on imbalanced class problems like PHU detection. Naïve Bayes models are very easy to train, so a fine-tuned parameter search was performed to find more than 1 robust CNB for use in the stacking ensemble [18].

Second, we integrated a random forest (RF) classifier in the ensemble model. An RF model is a meta-estimator that fits numerous decision tree classifiers on subsets of data features and averages results (ie, polls) to improve performance [19]. Decision trees, and by association RFs, are useful in several applications due to their explainability and ease of training. Decision trees do not require normalization and can accept categorical and numerical variables; however, a shortcoming of decision trees is their difficulty with generalization. Imprecise selection of hyperparameters will make the RF tree overly complex resulting in poor performance when facing unseen patterns [19]. Since RFs are an estimator built by decision trees, many of the parameters are carried over, although additional parameters are available for the sampling and final averaging with the RF [19].

All applicable parameters of an RF were varied through a random cross-validated grid search, but a few most notably contributed to overall performance and generalizability. These parameters include number of estimators, maximum depth, minimum samples to split, minimum samples at leaf, maximum number of features, and class weight. The number of estimators is the count of how many decision trees should be fitted to make up the RF [19]. Increasing the number of estimators typically increases generalizability but must be monitored for computational complexity. Maximum depth fixes the maximum number of levels that each tree can have, which is critical in generalizability [19]. If not set, the tree is continued until each leaf is pure, meaning the tree could learn the pattern of a single person in this population, which is not extensible to unseen populations. Minimum samples to split sets the minimum number of samples at the time of a split, ensuring that each leaf has at least n–1 samples. Minimum samples at leaf is very similar to minimum samples to split but controls samples at the leaf level. In this study, minimum samples at leaf was used to ensure edge cases (ie, unique PHU patterns) were still appropriately populated with training samples. Maximum number of features describes the method used to generate each tree which in certain use cases, taking the square root or log of the total number of features, can increase an RF’s performance [19].

Class weight is the most important RF parameter for performance, although setting it can negatively impact generalizability [19]. This parameter adjusts the prior weight on the positive class, which is important for imbalanced classes, and it pushes the decision tree fits to focus more closely on the minority class, making it more robust to edge cases. Since this model was designed to detect PHUs, favoring minority instead of majority class performance was key. Using specific class weights forced the decision trees to allow for a degradation in classifying non-PHUs in favor of an increase in PHU classification. Two RF models were selected from a random search cross-validation of parameters for use in the stacking ensemble. The final stacking ensemble model integrated the CNB and RF models into one predictive model.

The final ensemble model used an 80/20 split for training and testing of the data. We performed a 5-fold cross-validation on hyperparameter search and recursive feature elimination.

#### Performance Metrics

Typically, positive and negative class performance are assessed equally using a metric such as F1 score. In this study, as the PHU versus non-PHU classes are unequal and the positive class would constitute an infrequent occurrence, only the positive class metrics were considered key for performance improvement. Therefore, we measured PPV and sensitivity metrics to assess performance of all models (ie, individual models and ensemble model). Both performance metrics describe the classification results for the positive class (ie, PHUs). PPV is the proportion of positive classifications that are truly PHUs. Sensitivity is the proportion of PHUs who were classified as positive.

An important consideration in any machine learning algorithm evaluation is the balance among metrics. A simple way to find an appropriate balance is to change the threshold for classification. Choosing the appropriate threshold can be difficult for health care scenarios due to the risk of incorrect classification for an individual who needs treatment (ie, false negatives). Conversely, classifying too many healthy individuals at risk could overwhelm the resources available for interventions (ie, false positives). To address this issue, we calculated and then plotted sensitivity and PPV for 50 trials at thresholds spaced evenly .05 apart. We then calculated the discrimination threshold for the ensemble model to choose the optimal threshold of the PPV versus sensitivity metrics.

Finally, we compared the PPV and sensitivity of select individual models, which achieved at least 40% performance in both metrics, with the ensemble methodology. The individual models included a logistic regression, the Johns Hopkins ACG model (out-of-box and with no further training) [13], and a standalone RF model. The ensemble model included a stacking ensemble with multiple layers combining CNB and RF models.

All analyses, including descriptive analysis, individual modeling, and ensemble approach, were performed in R (version 3.5.1, R Foundation for Statistical Computing). We used Python pandas and scikit-learn for all modeling pipeline efforts (eg, data cleaning, filtering, hyperparameter search, feature selection, and RF model). We used Python ML Ensemble for the ensemble model [20]. We used Python Yellowbrick library to visualize the classification threshold of sensitivity versus positive predictive values. We used the Johns Hopkins ACG system to produce the ACG output and measure the ACG model’s performance [13].

## Results

### Descriptive Analyses

The study population comprised 165,595 unique patients including 8359 (5.1%) PHUs (Table 1). The PHU population’s average age was more than twice that of the non-PHU population (38.51 years vs 18.79 years). PHUs included fewer males (2735/8359, 32.7%) than non-PHUs (69,683/155,862, 44.7%). As expected, PHUs had more utilization than non-PHUs (1567/8359, 18.7% vs 3891/155,862, 2.5% for inpatient visits and 8332/8359, 99.7% vs 152,199/155,862, 97.3% for outpatient visits, respectively).

**Table 1:** Specification of the study populations (n=165,595).

			Overall study population (n=165,595)		Non-PHU^a^ population (n=155,862)		PHU population (n=8359)
**Age (years), mean (SD)**			19.85 (17.45)		18.79 (16.82)		38.51 (18.01)
	0-17, n (%)	101,264 (61.2)		99,352 (63.7)		1459 (17.5)	
	18-64, n (%)	63,260 (38.2)		55,666 (35.7)		6730 (80.5)	
	65+, n (%)	1037 (0.6)		844 (0.5)		170 (2.0)	
Sex (male), n (%)			72,974 (44.1)		69,683 (44.7)		2735 (32.7)
**Race, n (%)**							
	White	41,492 (25.1)		38,762 (24.9)		2457 (29.4)	
	Black	54,207 (32.7)		50,993 (32.7)		2879 (34.4)	
	Other^b^	149 (0.1)		143 (0.1)		6 (<0.1)	
	Missing^c^	69,747 (42.1)		65,964 (42.3)		3017 (36.1)	
**Inpatient visits, n (%)**							
	0	160,035 (96.6)		151,971 (97.5)		6792 (81.3)	
	1-5	5430 (3.3)		3866 (2.5)		1500 (17.9)	
	6-10	77 (<0.1)		20 (<0.1)		54 (0.6)	
	11+	19 (<0.1)		5 (<0.1)		13 (0.2)	
**Outpatient visits, n (%)**							
	0	3720 (2.2)		3663 (2.4)		27 (0.3)	
	1-5	96,122 (58.0)		94,138 (60.4)		1234 (14.8)	
	6-10	33,996 (20.5)		32,317 (20.7)		1428 (17.1)	
	11+	31,723 (19.2)		25,744 (16.5)		5670 (67.8)	

^a^PHU: persistent high utilizer.

^b^Members of known race/ethnicity not equal to Asian, Hispanic, White, or Black.

^c^Members with empty values for race.

### Ensemble Model

After tuning the ensemble layers, the best-performing ensemble model included 3 input layers and 1 prediction layer. The final ensemble model included 2 input layers of CNB and 1 layer of an RF model. The prediction layer was an RF model. The model included the following variables: race (ie, Black, White, other), age (as of 2013), sex, days of inpatient hospitalization in 2013, emergency department visit count in 2013, psychotherapy services in 2013, outpatient visit count in 2013, all-cause inpatient hospitalization count in 2013, frailty flag for older adults, 87 most frequent Johns Hopkins ACG diagnostic comorbidities (ie, EDCs [13]), all Johns Hopkins ACG medication grouping (ie, RxMGs [13]), and ACG-derived care coordination risk scores [13] (ie, likely coordination issue, possible coordination issue, unlikely coordination issue). These variables are generated by and included in the John Hopkins ACG risk stratification models, which are widely used for PHM efforts [13]. The stacking ensemble had full feature propagation throughout the layers to allow each model access to all data attributes while gaining classification scores from previous layers. The most performant models were selected for use in the stacking ensemble.

### Model Performance Evaluation

**Figure 3:**
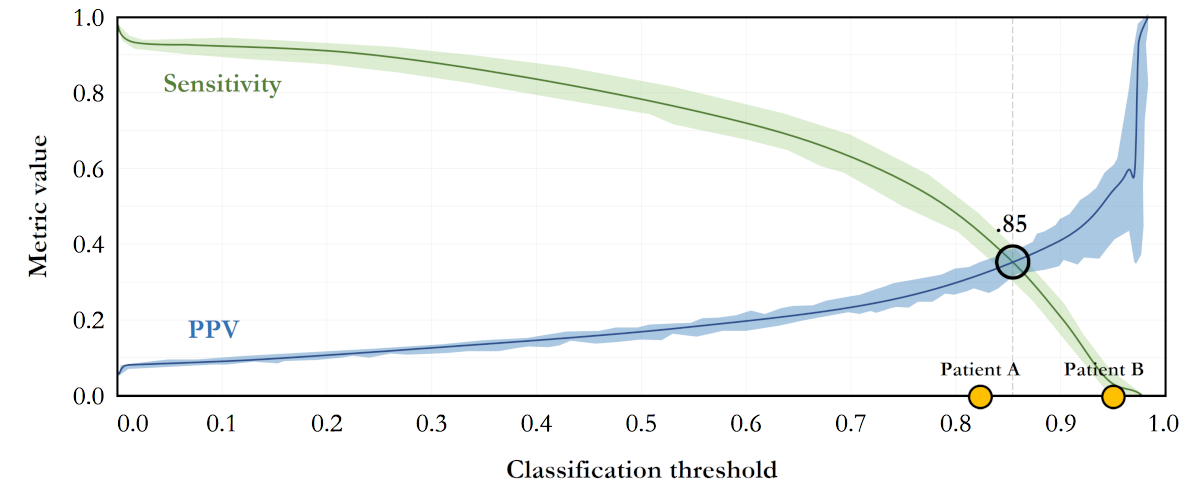
Classification threshold of sensitivity versus positive predictive value (PPV): patient A: incorrectly classified as normal (risk score=82%) and patient B: correctly classified as a persistent high utilizer (risk score=97%).

Figure 3 depicts the discrimination threshold plot for a sample decision tree of the ensemble model. The plot conveys the importance of the threshold choice and depicts the tradeoff between PPV and sensitivity. As shown in the figure, patients A and B, both of whom are PHUs, will be identified differently by the model depending on the chosen threshold between PPV and sensitivity. By testing the trained model on these 2 patients, a risk score is generated for each. These risk scores can be compared to any classification threshold. Depending on which side of the threshold the risk scores lie, the model classified whether patient A, B, or both are PHUs or non-PHUs.

The central line in Figure 3 represents the median value for each metric, and the bands represent the variability from the 10th to 90th percentiles. Two important observations about the threshold plot are (1) the typical classification threshold of .50 is not ideal probably due to the imbalanced classes and (2) equally weighting sensitivity and PPV at a threshold of .85 may not be appropriate to classify enough PHUs correctly. Patients A and B in Figure 3 have different classification outcomes and therefore interventions due in part to an arbitrary threshold.

To replicate the same level of optimality across all models, we used the 95th percentile threshold limit for each model. The absolute cutoff points were slightly different across models with ensemble having an absolute cutoff threshold of .258, RF .224, logistic regression .230, and the ACG model a cutoff of .226. Negative predictive value (NPV) and specificity were also assessed, but performance in these metrics was high (ie, averaging 97% and 99% for NPV and specificity, respectively) and did not vary significantly between models due to the large size and variability of the negative class (ie, non-PHUs).

### Performance Comparison

The stacking ensemble method achieved a sensitivity of 49.0% and PPV of 50.3%. The ensemble model resulted in a 5%+ increase in both PPV and sensitivity for predicting PHUs over other individual methods such as logistic regression, RF model, and the ACG model (Table 2). As shown in Table 2, the individual RF was the highest performing nonensemble technique. Table 2 also includes the optimal parameters used in the stacking ensemble (eg, CNB and RF parameters such as alpha, maximum depth, and minimum sample splits). The final ensemble model also produced an NPV of 97.4%, specificity of 97.3%, and F1 of 49.1% for PHUs and 97.4% for non-PHUs (not shown in Table 2). The area under the curve of the ensemble model reached .921; however, comparison of areas under the curve between models was considered not valuable due to the large imbalance of PHUs versus non-PHUs, hence limiting the performance measure comparison to PPV and sensitivity of the models.

**Table 2:** Model fit statistics for predicting persistent high utilizer status.

Model	Parameter tuning	Sensitivity, %	PPV^a^, %
Stacking ensemble Layer 1: CNB^b^ Layer 2: CNB Layer 3: RF^c^ Prediction layer: RF Feature propagation	CNB1 𝛼=.70, fit prior, norm CNB2 𝛼=.15, fit prior RF1 200 estimators 400 max^d^ depth 5 min^e^ samples split 0.01% min samples Leaf auto max features class weight=0.842 RF2 100 estimators 350 max depth 2 min samples split 0.01% min samples Leaf class weight=1.0	49.0	50.3
RF	300 estimators 500 max depth 20 min samples split 0.01% min samples leaf	48.4	47.2
JHU-ACG^f^	ACG^g^ system probability of PHU^h^	44.7	44.1
Logistic regression	Based on 241 parameters (ie, diagnoses and medications)	46.8	46.1

^a^PPV: positive predictive value.

^b^CNB: complement naïve Bayes.

^c^RF: random forest.

^d^max: maximum.

^e^min: minimum.

^f^JHU-ACG: ACG predictive model with no local tuning.

^g^ACG: adjusted clinical group.

^h^PHU: persistent high utilizer.

## Discussion

### Principal Findings

Persistent high utilizers (PHUs) are defined as patients who consistently stay in the highest deciles of health care costs or utilization across multiple years [4-12]. Risk stratification efforts strive to better identify and manage PHUs so that scarce health care resources can be better allocated. Nonetheless, predicting who becomes a PHU is often challenging, partly because PHUs are uncommon [4,6,9-11]. Past studies have attempted to improve the prediction of PHUs in various populations; however, those predictions have either suffered from high false negative/positive rates or have been limited in scope [4,6,9-11]. In this study, to address the methodological complexity in predicting PHUs, we evaluated the benefit of an ensemble approach to balance the sensitivity and specificity of predicting PHUs.

Our results show that ensemble methodology can be effectively used to improve both sensitivity and PPV of predicting PHUs. The ensemble model developed in this study included 2 layers of CNB and 1 prediction layer of RF, which can be converged rather quickly. We achieved a sensitivity and PPV of 49.0% and 50.3%, respectively, using the ensemble model. In comparison to the best alternative performing model, which was the standalone RF, the ensemble model improved the sensitivity by 0.6 and PPV by 3.1 absolute percentage points, which represents a 1.2% and 6.6% relative improvement in sensitivity and PPV, respectively. Moreover, standalone RF models are prone to overfitting and often lack generalizability to other populations. The ensemble model was also superior compared to traditional logistic regression and the more established (ACG) models [13]. The ensemble model improved the sensitivity and PPV of predicting PHUs by 2.2 and 4.2 absolute percentage points (ie, 4.7% and 9.1% relative improvement) compared to the traditional logistic regression and by 4.3 and 6.2 absolute percentage points (ie, 9.6% and 14.1% relative improvement) when compared to the ACG model [13].

Several studies have examined the use of traditional methods in predicting PHUs; however, models developed in these studies have often generated low PPV rates or showed limited generalizability. For example, in a study of an employer-based health plan, using commercial claims data, a logistic regression model achieved a sensitivity of 80% but PPV of 19% to predict PHUs among the health plan enrollees [6]. In another study aiming to predict PHUs, using diagnostic and medication information extracted from claims data, a regression model achieved a sensitivity of 46.7% and PPV of 57.2%; however, the study population was limited to patients aged 18 to 62 years, hence limiting generalizability to other populations [4]. Several studies have used regression models to control for underlying demographic and clinical variables and measure the residual differences such as cost, behavioral health, and social determinants of health variables between PHU and non-PHU populations [7,8]. These studies, however, have not published the performance of these regression models in predicting PHUs.

A few studies have assessed the value of machine learning methods in predicting PHUs. In a study of a statewide Medicaid population, demographics, diagnostics, and medication information were used to predict costs associated with PHUs. The study compared multiple models including linear regression, regularized regression, gradient boosting machine, and recurrent neural networks, but the study did not generate comparable predictive measures as these models did not predict PHU status [9]. Another study applied penalized regression, support vector machine, and extreme gradient boosting against claims data to predict PHUs among patients from an academic medical center. The study achieved high sensitivity rates ranging from 72.7% to 78.7%; however, the (recalculated) PPV ranged from 18.6% to 19.8% [10]. Among the machine learning studies targeting PHUs, only one study compared an ensemble methodology (using RFs) to other methods (eg, linear regression, decision tree regression) [11]. This study, however, predicted cost of PHUs and was limited to patients with schizophrenia, hence limiting its generalizability to the broader population of patients.

Despite the promising findings of past studies in predicting PHUs, their results cannot be accurately compared to our ensemble model as each study used a slightly different definition of PHU. Some studies have defined PHUs as patients in the top 5% of cost over 2 years [4], while other studies have set the bar at 10% or 20% of cost over longer periods of time [6,7]. Future research should attempt to harmonize the definition of PHUs to make the comparison of PHU populations across different populations and health plans feasible. Additionally, harmonization of the PHU definition can facilitate the performance measurement and comparison of PHU predictive models across different health care settings.

Balancing the sensitivity and PPV of PHU predictions is key in operationalizing such models in PHM efforts. Indeed, given the infrequency of PHUs in the total population of patients, a balanced sensitivity and PPV ratio will play an important role in the management of limited resources for PHUs. In our study, the improvement of model performance compared to the traditional models corresponds to approximately 84 additional PHUs being classified correctly in the test set of 1672 true PHUs. These 84 patients would not have been reviewed for potential proactive interventions by a care manager if tested by a traditional method.

In this study, we chose to report classification performance at the balanced precision and recall scores (50/50) to highlight optimal performance in both metrics simultaneously. In specific PHM use cases, it may be desirable to select a lower classification threshold and more patients for care or intervention consideration, even if their individual risk score is lower. In large-scale PHM use cases, cost of considering many patients may be too high and a higher classification threshold is to be selected to only manage the most at-risk patients. Hence, individual population health programs may chose different balances of precision versus recall for models predicting PHUs.

Our study showed that machine learning has a performance advantage over traditional statistical models. Ultimately, improved performance will come from more advanced ensemble methods coupled with continually improving robustness of feature analysis, which together are the keys to significantly increased performance. Model performance could benefit from subpopulation training by reducing the large and variable parameter space for classification. Thus, developing custom groupings of clinical features associate with PHU patients (versus non-PHUs) can potentially advance predictive models of PHUs. For example, clinical groupings identified by unsupervised machine learning techniques (such as latent class analysis) has shown value in improving predictive models of PHUs [21].

Value-based health care providers are increasingly using risk stratification tools to manage their patient populations [22]. Providers often use local electronic health records (EHRs) instead of insurance claims to risk stratify patients and predict PHUs [23-25]. Although advances have been made in using unique EHR data to improve risk prediction using prescription data [26-28], vital signs [29,30], laboratory results [31], and free-text analysis [32,33], quality of EHR data remains a major challenge in this process [34]. Using machine learning models, such as the ensemble models, can potentially help providers address some of these deficiencies and improve the prediction of PHUs using EHR data [35,36]. Future studies should investigate the usability of machine learning models in enhancing EHR-based PHU predictions and its implication on improving the wider population-level health outcomes [37].

### Limitations

Our study has several limitations. First, the results of our ensemble approach and the improvement of the PHU prediction may not generalize to other populations (eg, older adults), different settings (eg, inpatient only), or alternative data sources (eg, EHRs). Future research should explore the use of ensemble methodology in new populations and settings using alternate data sources. Second, the current definition of PHU may not be consistent with the operational definition in all PHM. We used a specific definition for PHU (ie, percentile of cost and time period), but that definition may not fit all populations. The risk stratification research community should harmonize the definition of PHU so predictive models of PHUs can be compared accurately to increase their generalizability. Third, we only used demographics, diagnosis, and medications in our prediction models. Past research has shown the value of social determinants of health in improving the prediction of health care utilization [38-42]. Future research should investigate the value of the ensemble model in improving predictive models of PHU that incorporate social data. Finally, the ensemble methodology uses an approach that complicates the explanation of a prediction, and thus the operational use of such models in clinical and PHM settings should be further studied.

### Conclusion

A small segment of the patient population uses most of the health care services over extended periods. We used an ensemble model, a machine learning approach that combines multiple modeling techniques, to simultaneously improve the sensitivity and PPV of predicting PHUs using claims data. Future studies should investigate the value of machine learning techniques in predicting PHUs in other health care settings with potentially different underlying populations and different data sources (eg, EHR data).

## Abbreviations

ACG: Adjusted Clinical Group
CNB: complement naïve Bayes
EDC: expanded diagnostic cluster
EHR: electronic health record
NPV: negative predictive value
PHM: population health management
PHU: persistent high utilizer
PPV: positive predictive value
RF: random forest
RxMG: Rx-defined morbidity group
